# In Situ Gel Incorporating Disulfiram Nanoparticles Rescues the Retinal Dysfunction via ATP Collapse in Otsuka Long–Evans Tokushima Fatty Rats

**DOI:** 10.3390/cells9102171

**Published:** 2020-09-25

**Authors:** Saori Deguchi, Fumihiko Ogata, Mizuki Yamaguchi, Misa Minami, Hiroko Otake, Kazutaka Kanai, Naohito Kawasaki, Noriaki Nagai

**Affiliations:** 1 Faculty of Pharmacy, Kindai University, 3-4-1 Kowakae, Higashi-Osaka, Osaka 577-8502, Japan; 2045110002h@kindai.ac.jp (S.D.); ogata@phar.kindai.ac.jp (F.O.); 2033420005s@kindai.ac.jp (M.Y.); 2033420004w@kindai.ac.jp (M.M.); hotake@phar.kindai.ac.jp (H.O.); kawasaki@phar.kindai.ac.jp (N.K.); 2 Department of Small Animal Internal Medicine, School of Veterinary Medicine, University of Kitasato, Towada, Aomori 034-8628, Japan; kanai@vmas.kitasato-u.ac.jp

**Keywords:** nanoparticles, disulfiram, diabetic retinopathy, electroretinogram, diethyldithiocarbamate

## Abstract

We attempted to design an ophthalmic in situ gel formulation incorporating disulfiram (DIS) nanoparticles (Dis-NPs/ISG) and demonstrated the therapeutic effect of Dis-NPs/ISG on retinal dysfunction in 15-month-old Otsuka Long–Evans Tokushima Fatty (OLETF) rats, a rat model of diabetes. The DIS particles were crushed using a bead mill to prepare the nanoparticles, and the Dis-NPs/ISG was prepared using a combination of the DIS nanoparticles and an in situ gelling system based on methylcellulose (MC). The particle size of the Dis-NPs/ISG was 80–250 nm, and there was no detectable precipitation or aggregation for 1 month. Moreover, the Dis-NPs/ISG was gelled at 37 °C, and the drug was delivered into the retina by instillation. Only diethyldithiocarbamate (DDC) was detected in the retina (DIS was not detected) when the Dis-NPs/ISG was instilled in the right eye, and the DDC levels in the right retina were significantly higher than those in the left retina. In addition, the retinal residence time of the drug was prolonged by the application of the in situ gelling system, since the DDC levels in the retinas of rats instilled with Dis-NPs/ISG were higher than those in DIS nanoparticles without MC. Furthermore, repetitive instillation of the Dis-NPs/ISG attenuated the deterioration of electroretinograms (ERGs) in 15-month-old OLETF rats by preventing the collapse of ATP production via excessive nitric oxide and recovered the decrease in retinal function. These findings provide important information for the development of novel therapeutic approaches to diabetic retinopathy.

## 1. Introduction

It is predicted that the proportion of patients with diabetes mellitus (DM) will increase to 4.4% in 2030, and the management and prevention of DM are now global public health problems [1,2]. DM patients develop a prothrombotic state involving impaired coagulation, platelet dysfunction, and endothelial dysfunction [3,4] and the onset of diabetic complications, such as microvascular complications, nephropathy, and neuropathy. In particular, diabetic retinopathy (DR) is an important hallmark of DM. DR is one of the main microvascular complications in DM patients and is characterized by cell death in retinal capillaries, glial and neuronal dysfunction, vessel occlusion, vascular leakage, and visual impairment, resulting in blindness [5].

The pathogenic mechanisms of DR were investigated using animal models of DM, and Ins2^Akita^ mice, a mouse model of type 1 diabetes, and rats with streptozocin-induced diabetes (STZ rats) have been used as animal models to investigate DR [6,7]. However, the pathophysiology in these models of DM is different from that of type 2 DM. However, Otsuka Long–Evans Tokushima Fatty (OLETF) rats develop type 2 DM with a metabolic syndrome and exhibit hyperglycemia, resistance to insulin, and pathological characteristics resembling human type 2 DM at 5–10 months of age [8,9,10,11,12]. Moreover, it was reported that the morphologic changes in the retinal capillaries and retinal ultrastructures of 14-month-old OLETF rats were similar to those of DM patients [13,14]. The changes in electroretinograms (ERGs) of OLETF rats also resemble those of DR patients [15]. Thus, OLETF rats have potential as a model of DR. We also reported that long-term hyperglycemia induced a glycation reaction, resulting in the enhancement of nitric oxide (NO) production via inducible NO synthase (iNOS) in the retinas of OLETF rats. Excess NO binds to cytochrome c oxidase (CCO), and the CCO–NO bond reversibly prevented CCO function by reducing the affinity of the enzyme for oxygen [16]. Then, the CCO dysfunction caused a decrease in the ATP levels in the retinas of 15-month-old OELTF rats [16].

Disulfiram (DIS) is a dimer of diethyldithiocarbamate (DDC) and has long been used to treat alcoholic syndrome. The DIS is a potent NOS inhibitor and a radical scavenger, and our previously studies showed that the converted DDC, after the oral administration of DIS, attenuated the iNOS in the retina and prevented the collapse of ATP production in the retinas of 15-month-old OELTF rats [16]. Furthermore, we reported that transcorneal penetration in DDC solution was low, although DIS can be applied as a prodrug in an ophthalmic DDC formulation to penetrate the cornea [17]. It is possible that the delivery of DIS into the retina has the ability to improve retinal dysfunction in DR.

The blood–retinal barrier, corneal barrier, and tear film barrier restrict the entry of drugs into the eye. Due to the structure and complex physiology of the eye, it is much harder to deliver drugs to the posterior segment [18], and instillation of traditional ophthalmic formulations cannot provide and maintain an adequate drug concentration in the retina [19,20]. There is an urgent need to develop an ingenious strategy for delivering a drug into a retina by instillation. To overcome these shortcomings of traditional ophthalmic formulations, ophthalmic drug delivery systems (DDSs), such as nanosuspensions, dendrimer in situ gels, nanocarriers, dendrimer nanospheres, and liposomes, have been studied extensively [21,22,23,24,25]. Gel systems that are instilled as a solution and transform into a gel at the ocular surface are called in situ gelling systems, and in situ gel used as in situ gelling systems usually undergo reversible sol–gel phase transitions. In order to further increase the ocular bioavailability (*BA*), we attempted to combine nanoparticles and an in situ gelling system. We also reported that a combination of nanoparticles and an in situ gelling system using methylcellulose (MC) increased the ocular *BA* [26,27]. These high ocular *BA*s may enable drug delivery to the retina.

In this study, we attempted to design an ophthalmic in situ gel formulation incorporating DIS nanoparticles (Dis-NPs/ISG) and investigated whether the Dis-NPs/ISG delivered the drug into the retina by instillation. Moreover, we demonstrated the therapeutic effect of Dis-NPs/ISG on retinal dysfunction in 15-month-old OLETF rats.

## 2. Materials and Methods

### 2.1. Animals

Male 15-month-old control Long–Evans Tokushima Otsuka rats (normal rat) and OLETF rats were obtained from Hoshino Laboratory Animals, Inc. These animals were housed at 25 °C (room temperature) and freely fed a commercial diet (CE-2, Clea Japan Inc., Tokyo, Japan) and water. All procedures were approved by Kindai University on 1 April 2013 (project identification code KAPS-25-003) and were performed in accordance with the guidelines of the Association for Research in Vision and Ophthalmology on the use of animals in research.

### 2.2. Chemicals

Isoflurane, benzophenone, mannitol (D-mannitol), a Cholesterol E-Test Kit, and DDC were purchased from Wako Pure Chemical Industries, Ltd. (Osaka, Japan). DIS powder and 2-hydroxypropyl-β-cyclodextrin (HPCD) were kindly donated by Ouchi Shinko Chemical Industrial Co., Ltd. (Tokyo, Japan) and Nihon Shokuhin Kako Co., Ltd. (Tokyo, Japan), respectively. A Mitochondrial Isolation kit, a Cytochrome c Oxidase Assay kit, and an ATP Bioluminescent Assay kit were purchased from Sigma Aldrich Japan (Tokyo, Japan). A Bio-Rad Protein Assay kit was provided by BIO-RAD (California, USA). We obtained 0.5% phenylephrine and 0.5% tropicamide from Santen Pharmaceutical Co., Ltd. (Osaka, Japan). An ELISA Insulin Kit and an RNA PCR Kit (AMV Ver 3.0) were purchased from Morinaga Institute of Biological Science Inc. (Kanagawa, Japan) and Takara Bio Inc. (Shiga, Japan), respectively. Methylcellulose type SM-4 (MC) was obtained from Shin-Etsu Chemical Co., Ltd. (Tokyo, Japan). Pentobarbital was provided by Sumitomo Dainippon Pharma Co., Ltd. (Toyo, Japan). Benzalkonium chloride (BAC), methanol, and acetonitrile was purchased from Kanto Chemical Co., Inc. (Tokyo, Japan). LightCycler FastStart DNA Master SYBR Green I was provided by Roche Diagnostics Applied Science (Mannheim, Germany). All other used chemicals were of the highest purity commercially available.

### 2.3. Preparation of DIS Solution

One percent DIS powder was added to a 10% HPCD solution containing 0.1% mannitol and 0.001% BAC and stirred for 24 h in the dark at 22 °C. After that, the solution was filtered using a 0.20 µm Minisart CE (Costar, Cambridge, MA, USA), and the filtrated solution (the DIS–HPCD inclusion complex) was used as DIS-sol. In the filtrate, the adsorption of DIS was not observed, and the pH was adjusted to 6.5.

### 2.4. Preparation of Ophthalmic Formulation Containing DIS Nanoparticles

The mixture containing 1% DIS powder (mean particle size: 97.5 ± 36.4 µm), 0.1% mannitol, 0.001% BAC, and 0.5% MC was placed in a zirconia mortar with a zirconia ball and milled at 100 rpm for 7 d at 4 °C using a stirrer for a magnetic mortar (AS ONE Corporation, Osaka, Japan). After that, the mixture was dispersed in purified water, and the dispersions were transferred to a tube with zirconia beads (diameter: 0.1 mm) and milled with the Bead Smash 12 (5500 rpm, 30 s, 4 °C) 30 times. Then, the milled dispersions with zirconia beads were crushed using a Shake Master NEO (Bio Medical Science, Tokyo, Japan) at 1500 rpm and 4 °C for 1 h. The milled dispersions were used as Dis-NPs. Moreover, the Dis-NPs/ISG was prepared by adding 1.5% MC to Dis-NPs. Table 1 shows the compositions of the DIS formulations used in this study.

### 2.5. Measurement of DIS and DDC

Fifty microliters of sample was added to 100 µL of methanol containing 0.3 µg of benzophenone (internal standard) and filtered through a Chromatodisk 4A (pore size: 0.45 µm, Kurabo Industries Ltd., Osaka, Japan). Ten microliters of the filtrated solution was injected into an LC-20AT HPLC system using an SIL-10AF auto sampler (Shimadzu Corp., Kyoto, Japan). We used an Inertsil^®^ ODS-3 column (2.1 mm × 50 mm, GL Science Co., Inc., Tokyo, Japan), and the column temperature was set to 35 °C using a CTO-20A column oven (Shimadzu Corp., Kyoto, Japan). Here, 40% acetonitrile containing 0.1% trifluoroacetic acid was used as a mobile phase and the flow rate was adjusted to 0.25 mL/min, and the levels of DIS and DDC were detected at 215 nm in an SPD-20A UV detector (Shimadzu Corp., Kyoto, Japan).

### 2.6. Characteristics of the Ophthalmic DIS Formulations

The particle size distribution of the DIS powder was measured using a light microscope and an SALD-7100 laser diffraction particle size analyzer (Shimadzu Corp., Kyoto, Japan). The particle size distribution and the number of DIS nanoparticles were determined using the SALD-7100 and a NanoSight LM10 dynamic light scatterer (Quantum Design Japan, Tokyo, Japan). Moreover, the number of DIS nanoparticles was also measured using the NanoSight LM10. Atomic force microscope (AFM) images of DIS nanoparticles were provided by an SPM-9700 (Shimadzu Corp., Kyoto, Japan). A MCR302 rheometer with a CP50-1 (Anton Paar Japan, Tokyo, Japan) was used to measure the viscosities of the DIS at 20 and 37 °C. The unsolubilized DIS in the Dis-NPs and the Dis-NPs/ISG was separated by centrifugation (100,000 g) using a Beckman Optima^TM^ MAX-XP Ultracentrifuge (Beckman coulter, Osaka, Japan). The above-described HPLC method was used to measure the contents of solubilized and unsolubilized DIS and evaluate the solubility. The zeta potential in the DIS formulations was measured using a micro-electrophoresis zeta potential analyzer (model 502, Nihon Rufuto Co., Ltd., Tokyo, Japan).

### 2.7. Dispersibility of Ophthalmic DIS Formulations

The ophthalmic DIS formulations were stored in the dark at 20 °C for 1 month. Then, solution containing DIS was taken from 5 mm under the surface and used as a sample. The DIS contents in the provided samples were measured by the above-described HPLC method, and the contents were evaluated in terms of the dispersibility of DIS formulations. Moreover, the particle size and number of DIS nanoparticles in the samples were analyzed using the NanoSight LM10 to demonstrate the changes in particle states over time.

### 2.8. Measurement of DIS Content in the Retina

Thirty microliters of ophthalmic DIS formulation was instilled into the right eye of 15-month-old OLETF rats without anesthesia, and the rats were left for 0–100 min. Then, the rats were killed by injecting a lethal dose of pentobarbital and the retinas were removed. The collected retinas were homogenized in methanol on ice and centrifuged at 20,400 g for 15 min at 4 °C. The DIS and DDC concentrations in the supernatant were measured using the above-described HPLC method.

### 2.9. Corneal Toxicity

Thirty microliters of Dis-NPs/ISG was repeatedly instilled into the right eye of OLETF rats (14–15 months of age) for 1 month (twice a day, 9:00 a.m. and 7:00 p.m.). Then, the toxicity (wound area) was stained by 1% fluorescein (30 μL). A Topcon TRC-50X (Topcon, Tokyo, Japan) was used to monitor the corneal wound in each rat.

### 2.10. Evaluation of Parameters for DM

Thirty microliters of the vehicle and Dis-NPs/ISG were repeatedly instilled into the right eye of OLETF rats (14–15 months of age) for 1 month (twice a day, 9:00 a.m. and 7:00 p.m.). Then, blood was collected from the tail vein of 15-month-old OLETF rats that had fasted for 12 h without anesthesia. Plasma glucose and triglyceride levels were measured using an Accutrend GCT System (Roche Diagnostics, Mannheim, Germany). During the measurement of the insulin and total cholesterol levels, the collected blood was centrifuged at 20,400 g for 15 min at 4 °C, and the supernatant was used to measure the plasma insulin and cholesterol levels. The ELISA Insulin Kit and the Cholesterol E-Test Kit were used to analyze the insulin and cholesterol, respectively. All analyses were performed according to the manufacturer’s instructions.

### 2.11. Quantitative Real-Time RT-PCR

Thirty microliters of the vehicle and Dis-NPs/ISG were repeatedly instilled into the right eye of OLETF rats (14–15 months of age) for 1 month (twice a day, 9:00 a.m. and 7:00 p.m.). Then, the 15-month-old OLETF rats were killed by injecting a lethal dose of pentobarbital, and the retinas were removed. All of the RNAs were extracted from the retinas using the acid guanidium thiocyanate–phenol–chloroform extraction method. The OD_260_/OD_280_ values of all RNAs used were greater than 1.8. The RNA PCR Kit was used to perform the RT reaction, and the PCR reactions were performed using LightCycler FastStart DNA Master SYBR Green I. All reactions were performed according to the manufacturer’s instructions. The following primers were used: 5′-CGCTCCTGGAAGATGGTGAT-3′ for glyceraldehyde-3-phosphate dehydrogenase (GAPDH, GenBank accession No. NM_017008); 5′-GGAGAGATTTTTCACGACACCC-3′ and 5′-CCATGCATAATTTGGACTTGCA-3′ for iNOS (GenBank accession No. NM_012611); and 5′-ACGGCACAGTCAAGGCTGAGA-3′. The conditions for the PCR were set to 95 °C for 10 min, 50 cycles of 95 °C for 10 s, 60 °C for 10 s, and 72 °C for 5 s. The differences in the threshold cycles for iNOS and GAPDH were used to calculate the levels of mRNA expression in the rats.

### 2.12. Measurement of NO Levels

Thirty microliters of the vehicle and Dis-NPs/ISG were repeatedly instilled into the right eye of OLETF rats (14–15 months of age) for 1 month (twice a day, 9:00 a.m. and 7:00 p.m.). Then, the 15-month-old OLETF rats were killed by injecting a lethal dose of pentobarbital, and the retinas were removed. The collected retinas were homogenized in saline on ice, and the homogenates were centrifuged at 20,400 *g* for 15 min at 4 °C. The supernatants were used to measure NO levels. The NO_2_^−^ metabolite level is presented as the NO level. The ENO-20 NOx analyzer system was used to measure the NO_2_. The NO_2_^−^ was mixed with Griess reagent containing 0.25 g/L N-naphthylethylenediamine, 5 g/L sulfanilamide, and 1.25% HCl and incubated at 35 °C in a column oven. Then, the absorbance of the colored product dye at 540 nm was measured using an NOD-10 spectrophotometer (Eicom, Kyoto, Japan). The NO_2_^−^ levels are presented as nmol/mg of protein. The Bio-Rad Protein Assay Kit was used to measure the protein levels in the retinas of 15-month-old OLETF rats according to the manufacturer’s instructions.

### 2.13. Measurement of CCO Activity

Thirty microliters of the vehicle and Dis-NPs/ISG were repeatedly instilled into the right eye of OLETF rats (14–15 months of age) for 1 month (twice a day, 9:00 a.m. and 7:00 p.m.). Then, the 15-month-old OLETF rats were killed by injecting a lethal dose of pentobarbital, and the retinas were removed. The mitochondria in retina were isolated using the Mitochondrial Isolation kit according to the manufacturer’s instructions. The collected retinas were homogenized in isolation buffer containing 2 mg/mL albumin, 1 mM EDTA, 70 mM sucrose, 200 mM mannitol, and 10 mM HEPES. The homogenates were centrifuged at 600 g for 5 min at 4 °C, and the supernatants were centrifuged at 11,000 g for 10 min at 4 °C. The collected pellets were washed with isolation buffer 1. The resulting pellets were vortexed with 40 µL of buffer 2 containing 1 mM DTT, 2 mM K_2_HPO_4_, 5 mM sodium succinate, 0.08 mM ADP, 1 mM ATP, 200 mM mannitol, and 10 mM HEPES and used as the isolated mitochondria from retinas. The Cytochrome c Oxidase Assay kit was used to analyze the CCO activity and isolated mitochondria from the retinas of OLETF rats. The analysis was performed according to the manufacturer’s instructions. The CCO activity is presented as units/milligram of protein, and the protein level was measured by the above-described method.

### 2.14. Measurement of ATP Levels

Thirty microliters of the vehicle and Dis-NPs/ISG were repeatedly instilled into the right eye of OLETF rats (14–15 months of age) for 1 month (twice a day, 9:00 a.m. and 7:00 p.m.). Then, the 15-month-old OLETF rats were killed by injecting a lethal dose of pentobarbital, and the retinas were removed. The collected retinas were homogenized in 10 mM HEPES/KOH buffer (pH: 7.8) on ice, and the homogenates were centrifuged at 9100 g for 10 min at 4 °C. The supernatants were used to measure ATP levels. The ATP levels were analyzed using the ATP Bioluminescent Assay Kit according to the manufacturer’s instructions. A luciferin–luciferase reaction was detected by an AB-2200 luminometer (Atto Corporation, Tokyo, Japan). The ATP levels are presented as nmol/mg of protein, and the protein level was measured by the above-described method.

### 2.15. Measurement of ERG

Thirty microliters of the vehicle and Dis-NPs/ISG were repeatedly instilled into the right eye of OLETF rats (14–15 months of age) for 1 month (twice a day, 9:00 a.m. and 7:00 p.m.). Then, the 15-month-old OLETF rats were maintained in a completely dark room for 24 h. Afterward, the pupils in rats anesthetized with isoflurane were dilated by 0.5% phenylephrine and 0.5% tropicamide. ERG readings in the right eye of 15-month-old OLETF rats were performed according to our previous report and recorded by PuREC (Mayo, Aichi, Japan). The amplitude of the a-wave was measured from the baseline to the maximum a-wave peak, while the b-wave was measured from the maximum a-wave peak to the maximum b-wave peak. The oscillatory potential (OP) amplitudes were measured in the time interval between the a- and b-wave peaks. All procedures were performed under dim red light.

### 2.16. Statistical Analysis

F test and bartlett’s test were used to assess data normality, and one-way analysis of variance (ANOVA) followed by Dunnett’s multiple comparison test and Student’s *t*-test was used for statistical comparisons. The significance level was set at *p* < 0.05. The values are presented as the mean ± standard error of the mean (S.E.M).

## 3. Results

### 3.1. Design of the In Situ Gel Incorporating DIS Nanoparticles

**Figure 1:**
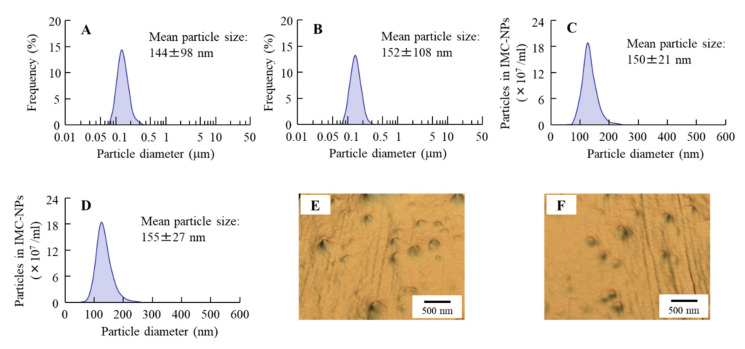
Size frequencies of disulfiram (DIS) particles in ophthalmic DIS formulations. **A** and **B**: Particle size of DIS nanoparticles (Dis-NPs) (**A**) and the in situ gel formulation incorporating DIS nanoparticles (Dis-NPs/ISG) (**B**) determined using the SALD-7100 laser diffraction particle size analyzer. C and D: Particle size of the Dis-NPs (**C**) and the Dis-NPs/ISG (**D**) determined using the NanoSight dynamic light scatterer. E and F: AFM image of the Dis-NPs (**E**) and the Dis-NPs/ISG (**F**) taken using the SPM-9700. The particle size of DIS in the Dis-NPs and the Dis-NPs/ISG was approximately 80–250 nm.

**Figure 2:**
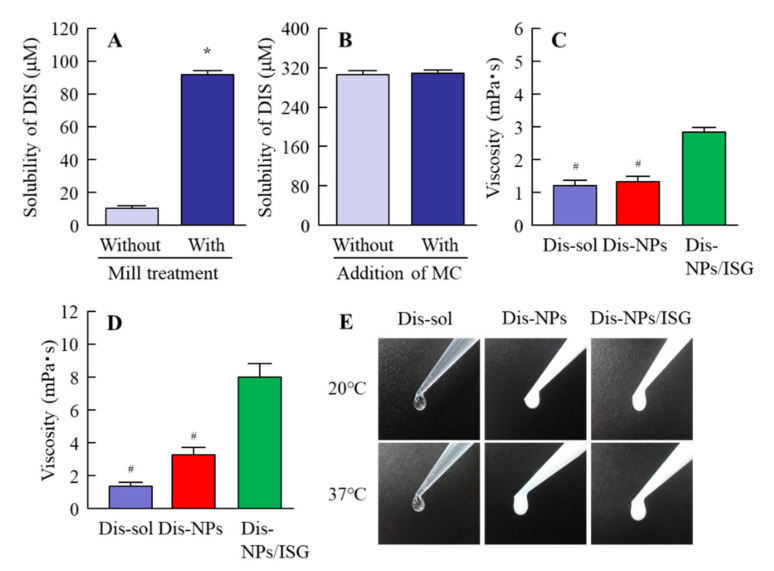
Solubility and viscosity of DIS in ophthalmic DIS formulations. **A**: Changes in DIS solubility in ophthalmic DIS formulations with or without the bead mill treatment. **B**: Effect of methylcellulose (MC) content on DIS solubility in ophthalmic Dis-NPs formulations. **C** and **D**: Viscosity of the disulfiram solution (Dis-sol), Dis-NPs, and Dis-NPs/ISG at 20 °C (**C**) and 37 °C (**D**). (**E**) Pictures of the Dis-sol, Dis-NPs, and Dis-NPs/ISG at 20 and 37 °C. The composition of the Dis-sol, the Dis-NPs, and the Dis-NPs/ISG is shown in Table 1. *n* = 9. *p < 0.05 vs. the group without mill treatment. ^#^*p* < 0.05 vs. the Dis-NPs/ISG for each category. Although the solubility of DIS in the group with MC and the group without MC was similar, the bead mill treatment enhanced the solubility of DIS. On the other hand, the Dis-NPs/ISG became gel at 37 °C, and the viscosity had increased.

**Figure 3:**
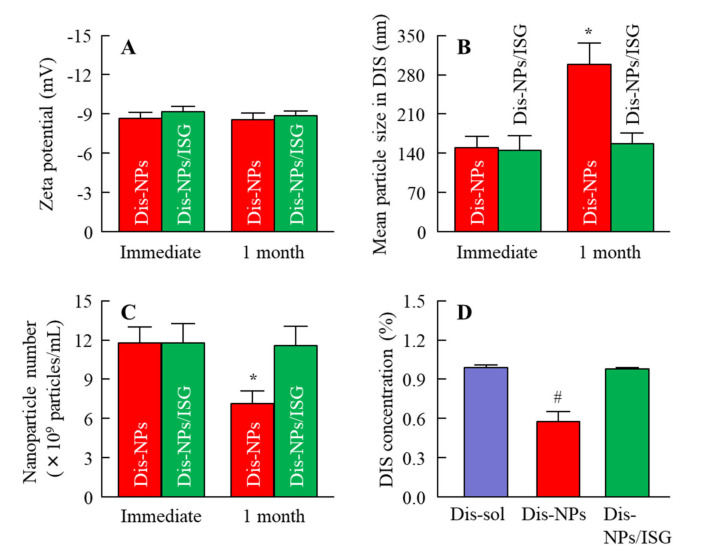
Changes in the zeta potential of, and the nanoparticles and dispersibility in, the ophthalmic DIS formulations 1 month after bead mill treatment. A–D: Zeta potential (**A**), mean particle size (**B**), number of particles (**C**), and dispersibility (**D**) of DIS in the ophthalmic DIS formulations. The composition of the Dis-sol, the Dis-NPs, and the Dis-NPs/ISG is shown in Table 1. *n* = 8. *p < 0.05 vs. the immediate group for each category. ^#^*p* < 0.05 vs. the Dis-sol. The zeta potential of the Dis-NPs and the Dis-NPs/ISG was similar. Aggregation and precipitation in the Dis-NPs was observed 1 month after preparation, since the particle size was increased, and the number of nanoparticles was decreased. In contrast to the results in Dis-NPs, the Dis-NPs/ISG remained stable 1 month after preparation.

We previously showed that the instillation of suspensions containing DIS nanoparticles can supply more DDC to the aqueous humor and lens than a conventional formulation (DIS-sol) [28,29]. However, the delivery of a sufficient amount of the drug to the retina by eye drops is difficult; so, we need to improve ophthalmic DDSs for retinal drug delivery. It has been shown that the ocular *BA* could be enhanced by the improvement of the corneal permeability time and the precorneal residence of the drug [30]. Therefore, in this study, we designed an in situ gel incorporating DIS nanoparticles (Dis-NPs/ISG). Figure 1 shows the size frequencies of Dis-NPs and the Dis-NPs/ISG. The particle size of DIS was decreased, and the size of DIS in DIS-NPs was 80–250 nm. Moreover, the added 1.5% MC did not affect the particle size, and the particle size in the Dis-NPs/ISG was also 80–250 nm. Figure 2 shows the solubility and viscosity of DIS in the Dis-NPs and the Dis-NPs/ISG. The solubility of the DIS with the bead mill treatment was higher than that of the DIS without the bead mill treatment (Figure 2A), and the Dis-NPs and Dis-NPs/ISG had similar solubility (Figure 2B). These results suggest that the drug’s solubility is increased by nanoparticulation and that the addition of MC does not change the solubility. The unsolubilized DIS in both the Dis-NPs and the Dis-NPs/ISG accounted for approximately 0.27% of the total DIS formulation (approximately 99.7% was solubilized DIS). It is known that the MC (SM-4) used in this study becomes gel at temperatures above 32–35 °C. Therefore, we measured the viscosity of the Dis-sol, Dis-NPs, and Dis-NPs/ISG at 20 and 37 °C. At 20 °C, the viscosity of the Dis-sol and the Dis-NPs was similar, although the viscosity was increased by the addition of MC. On the other hand, at 37 °C, the MC in the Dis-NPs and the Dis-NPs/ISG had gelled, and the viscosities of the Dis-NPs and the Dis-NPs/ISG had increased. The viscosity of the Dis-NPs and the Dis-NPs/ISG was 2.4- and 5.9-fold that of the Dis-sol at 37 °C, respectively. Figure 3 shows the zeta potential of, and the number of nanoparticles and dispersibility in, the Dis-NPs/ISG 1 month after preparation. In both the Dis-NPs and the Dis-NPs/ISG, there was no change in the zeta potential immediately and 1 month after preparation, and the zeta potential was approximately 8.8–9.2 mV. On the other hand, the dispersibility in the Dis-NPs/ISG was higher than that in the Dis-NPs. Both precipitation and aggregation were observed in the Dis-NPs 1 month after preparation. The size and number of nanoparticles in the Dis-NPs/ISG did not change 1 month after preparation, and there was no detectable precipitation or aggregation. These results show that the high viscosity caused by MC enhanced the dispersibility in the suspensions containing DIS nanoparticles.

### 3.2. Delivery of a Drug to the Retina by the Instillation of Ophthalmic DIS Formulations

**Figure 4:**
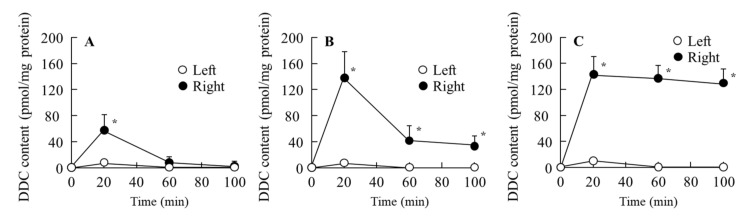
Changes in diethyldithiocarbamate (DDC) levels in the retinas of rats following the instillation of Dis-sol (**A**), Dis-NPs (**B**), and the Dis-NPs/ISG (**C**). The ophthalmic DIS formulations were instilled into the right eye of rats. The composition of the Dis-sol, the Dis-NPs, and the Dis-NPs/ISG is shown in Table 1. *n* = 4–8. *p < 0.05 vs. left for each category. Although DIS was not detected, the DDC was increased in the retinas instilled with DIS formulations. The DDC levels in the right retina were higher than those in the left retina in the used DIS formulations. Moreover, the DDC levels in the right retina of rats instilled with Dis-NPs were higher than those in the right retina of rats instilled with Dis-sol, and the retinal residence time of DDC was prolonged by the application of the in situ gelling system.

It is important to clarify how a drug is delivered to the retina by instillation of ophthalmic DIS formulations. Therefore, the changes in drug levels in the retina were measured. They are shown in Figure 4. The DIS was not detected in the retina after instillation in all three formulations (Dis-sol, Dis-NPs, and Dis-NPs/ISG) used in this study. On the other hand, only DDC was detected in the retina instilled with all three formulations. In the Dis-sol, the concentration of DDC peaked 20 min after instillation and then gradually decreased. The DDC level was almost undetectable 60 min after instillation (Figure 4A). The DDC levels in the retinas of rats instilled with Dis-NPs were higher than those in the retinas of rats instilled with Dis-sol. The DDC level was approximately 140 pmol/mg of protein 20 min after instillation (Figure 4B). Otherwise, the DDC levels in the retinas of rats instilled with Dis-NPs gradually decreased over the 20–100 min period after instillation. However, in the case of Dis-NPs/ISG, there was less of a decrease over the 20–100 min period after instillation, and 130–140 pmol/mg of protein of DDC was maintained (Figure 4B,C). In this study, we evaluated if repetitive instillation of Dis-NPs/ISG caused corneal toxicity (wound) using a Topcon TRC-50X. No toxicity (corneal wound) due to the Dis-NPs/ISG was observed when the Dis-NPs/ISG was repeatedly instilled into the right eye of OLETF rats for 1 month (twice a day, 9:00 a.m. and 7:00 p.m.).

### 3.3. Preventive Effect of the Dis-NPs/ISG on Retinal Disorders in OLETF Rats

**Figure 5:**
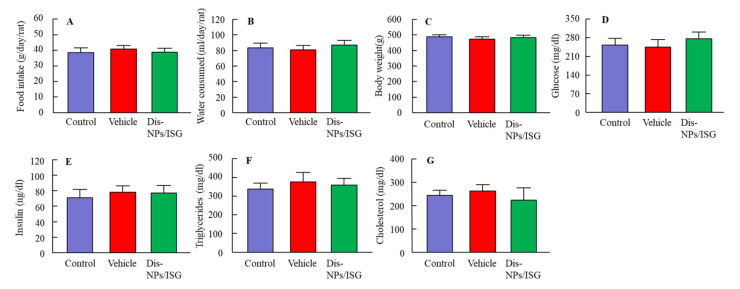
Effect of the Dis-NPs/ISG on food (**A**) and water (**B**) intake, body weight (**C**), plasma glucose (**D**), plasma insulin (**E**), plasma triglycerides (**F**), and plasma cholesterol (**G**) levels in the 15-month-old Otsuka Long–Evans Tokushima Fatty (OLETF) rats. The Dis-NPs/ISG was repeatedly instilled into the right eye of OLETF rats for 1 month (twice a day, 9:00 a.m. and 7:00 p.m.). Control, noninstilled OLETF rat; Vehicle, vehicle-instilled OLETF rat; Dis-NPs/ISG, Dis-NPs/ISG-instilled OLETF rat. *n* = 5–6. The repetitive instillation of Dis-NPs/ISG did not affect the food and water intake and some blood test values for diabetes mellitus (DM) in the OLETF rats.

**Figure 6:**
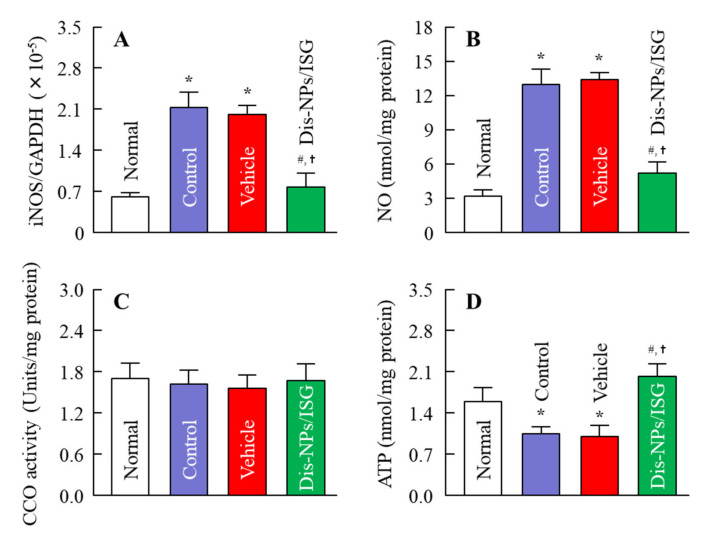
Effect of the Dis-NPs/ISG on inducible NO synthase (iNOS) mRNA (**A**), nitric oxide (NO) (**B**), cytochrome c oxidase (CCO) activity (**C**), and ATP levels (**D**) in the retinas of 15-month-old OLETF rats. The Dis-NPs/ISG was repeatedly instilled into the right eye of OLETF rats for 1 month (twice a day, 9:00 a.m. and 7:00 p.m.). Normal, noninstilled normal rat; Control, noninstilled OLETF rat; Vehicle, vehicle-instilled OLETF rat; Dis-NPs/ISG, Dis-NPs/ISG-instilled OLETF rat. *n* = 6–8. *p < 0.05 vs. normal for each category. ^#^*p* < 0.05 vs. control for each category. ^†^*p* < 0.05 vs. vehicle for each category. The repetitive instillation of the Dis-NPs/ISG prevented NO production via iNOS mRNA and attenuated the decrease in ATP levels in the retinas of 15-month-old OLETF rats.

**Figure 7:**
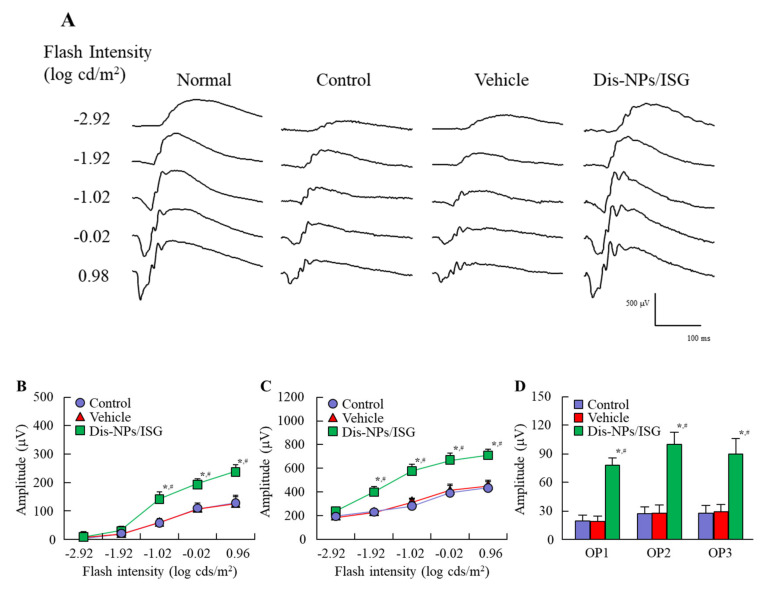
Typical traces of electroretinograms (ERGs) (**A**), a-wave amplitude (**B**), b-wave amplitude (**C**), and OP amplitude (**D**) in the right retina of 15-month-old OLETF rats repeatedly instilled with Dis-NPs/ISG. The Dis-NPs/ISG was repeatedly instilled into the right eye of OLETF rats for 1 month (twice a day, 9:00 a.m. and 7:00 p.m.). Normal, 4-month-old OLETF rat; Control, noninstilled OLETF rat; Vehicle, vehicle-instilled OLETF rat; Dis-NPs/ISG, Dis-NPs/ISG-instilled OLETF rat. *n* = 6–7. *p < 0.05 vs. control for each category. ^#^*p* < 0.05 vs. vehicle for each category. The repetitive instillation of Dis-NPs/ISG recovered the decrease in retinal function.

OLETF rats are a model of human DM. In this study, we used OLETF rats to evaluate the preventive effect of instillation of the Dis-NPs/ISG on retinal disorders. Figure 5 shows the changes in food intake, water consumption, body weight, and blood markers for DM in the 15-month-old OLETF rats repeatedly instilled with the Dis-NPs/ISG. The Dis-NPs/ISG was repeatedly instilled for 1 month (the 14th to 15th month of age). The OLETF rats showed the onset of DM with excessive food intake and water consumption. The repetitive instillation of the Dis-NPs/ISG did not affect the food and water intake and some blood test values for DM in the OLETF rats. Figure 6 shows the levels of the iNOS mRNA, NO, CCO activity, and ATP in the retinas of 15-month-old OLETF rats repeatedly instilled with Dis-NPs/ISG. The levels of iNOS mRNA and NO in the retinas of OLETF rats were significantly higher than those in the retinas of normal rats. The CCO activity in the retinas of OLETF rats was similar to that in the retinas of normal rats, and the ATP levels in the retinas of OLETF rats were significantly lower than those in the retinas of normal rats. On the other hand, the NO production via iNOS mRNA and the decrease in ATP levels in the 15-month-old OLETF rats were attenuated by the repetitive instillation of Dis-NPs/ISG. Furthermore, we observed changes in retinal function in 15-month-old OLETF rats with or without repetitive instillation of Dis-NPs/ISG (Figure 7). Typical traces of ERG were attenuated in the OLETF rats, and the a-wave amplitude, the b-wave amplitude, and the OP amplitude in OLETF rats were lower in comparison with normal rats. The repetitive instillation of Dis-NPs/ISG recovered the typical traces of ERG and the decreased a-wave amplitude, b-wave amplitude, and OP amplitude.

## 4. Discussion

RD is a retinal disease caused by the adverse effects of hyperglycemia, and, over time, retinal dysfunction leads to a loss of vision. In this study, we designed an ophthalmic in situ gel incorporating DIS nanoparticles (Dis-NP/ISG) and found that the instillation of Dis-NPs/ISG was able to deliver a sufficient amount of drug into the retinas of rats in comparison with traditional ophthalmic formulations. Moreover, we showed that the instillation of Dis-NPs/ISG attenuated the collapse of ATP production by CCO dysfunction via excessive NO and recovered the deterioration of ERGs in the retinas of OLETF rats.

DIS is a quaternary ammonium compound and a dimer of DDC. DIS has been used as an anti-alcoholism drug in the clinic, and its long-term use is very safe and well-tolerated with few side effects [31]. After oral administration and instillation, DIS is converted to DDC by the aldehyde dehydrogenase (ALDH) in the intestine and cornea [17]. The DDC converted from DIS inhibits NFκB, resulting in attenuation of iNOS expression and excessive NO production in the ophthalmic field [28,29,32]. In addition, our previous study showed that the inhibition of NO by DIS instillation attenuated the onset of lens opacity and an increase in intraocular pressure [28,29,32] and that the oral administration of DIS recovered retinal ATP dysfunction [16]. Taken together, our results suggest that the instillation of DIS could be useful for the prevention of ophthalmic diseases. On the other hand, it is difficult to deliver the drugs into the retina by means of instillation. Therefore, in this study, we attempted to prepare an ophthalmic in situ gel incorporating DIS nanoparticles (Dis-NPs/ISG), and investigated whether the instillation of the Dis-NPs/ISG delivered the drug into the retina.

First, we designed ophthalmic DIS formulations. We previously prepared the formulation for the DDC solution, the DIS/HPCD inclusion complex, and the ophthalmic formulation containing DIS nanoparticles (Dis-NPs) [28,29] and showed that the DDC solution could not penetrate the cornea, since DDC is classified as a hydrophilic drug. On the other hand, DIS, which is hydrophobic, was found to penetrate the cornea. The Dis-sol is able to prepare by the DIS/HPCD inclusion complex [33]. In addition, DIS can be miniaturized to nanosized particles using the bead mill method with 0.5% MC [28,29]. According to these previous results, we prepared Dis-sol and Dis-NPs in this study (Table 1). The particle size of DIS was decreased by the bead mill treatment with 0.5% MC, the particle size was 80–250 nm (Figure 1), and the solubility was increased (Figure 2). These results show that the solubility of DIS was enhanced by the reduction in the size of DIS particles to the nano range.

The MC (SM-4) formed a physically cross-linked hydrogel at approximately 32–35 °C. MC is frequently used as a gelling agent in the ophthalmic field [34], and the proposed in situ gelling system based on MC shows promise for sustained ocular drug delivery. We also presented a combination of the in situ gelling system and tranilast nanoparticles and reported that the concentration of MC used in the in situ gelling system has an optimum value and that the release of nanoparticles is suppressed when a high MC (3%) concentration is used [26]. Based on these results, in this study, we used MC to prepare the Dis-NPs/ISG and determined the optimal MC content in the Dis-NPs/ISG to be 1.5%.

The addition of MC did not affect the particle size and solubility, and the viscosity was enhanced. The viscosity of the Dis-NPs/ISG was 2.85 mPa∙s at 20 °C, the Dis-NPs/ISG gelled at 37 °C, and the viscosity of the Dis-NPs/ISG was 8.01 mPa∙s at 37 °C (Figure 2). Furthermore, aggregation and precipitation in the Dis-NPs were observed 1 month after preparation. Although the addition of MC enhanced the dispersibility, the particle size and number of nanoparticles in the Dis-NPs/ISG had not changed after 1 month (Figure 3). It was found that the increase in viscosity led to an enhancement of the dispersibility of the particles, and the viscosity of the Dis-NPs/ISG was higher in comparison with the Dis-NPs (Figure 2). Moreover, the zeta potential of the Dis-NPs/ISG was similar to that of the Dis-NPs. The high degree of viscosity may enhance the dispersibility in the Dis-NPs/ISG. On the other hand, the unsolubilized DIS accounted for 99.7% in the Dis-NPs/ISG, and the solubilized DIS accounted for 0.28% (Figure 2). These results show that most DIS nanoparticles in the Dis-NPs/ISG exist as unsolubilized DIS.

Next, we determined whether the in situ gels incorporating DIS nanoparticles affected the amount of drug delivered into the retina following instillation. Our previous reports using DIS-sol showed that the DIS was converted to DDC by the aldehyde dehydrogenase 3A1 (ALDH3A1), a sulfhydryl-rich protein, in the cornea, and released to the intraocular region [17]. In this study, only DDC was detected in the retina after the instillation of Dis-NPs and the Dis-NPs/ISG, and the DDC levels in the right retina (the instilled eye) were higher in comparison with those in the left retina (the uninstilled eye) (Figure 4). Moreover, the DDC levels in the right retinas of rats instilled with Dis-NPs were higher than those in the right retinas of rats instilled with Dis-sol, and the retinal residence time of DDC was prolonged in the Dis-NPs/ISG (Figure 4). Taken together, it was suggested that the Dis-NPs/ISG provided the gel to the surface of the cornea and that the DIS in gel was absorbed by the cornea and the drug was released in the eye. In the process of corneal penetration, the DIS was converted to DDC, and the converted DDC was delivered into the retina, and the DIS locally affected the retina after the instillation. In addition, the in situ gels incorporating DIS nanoparticles were found to have the ability to effectively deliver the drug to the retina.

The selection of the experimental animal is very important to evaluate the therapeutic effect of Dis-NPs/ISG on retinal dysfunction in DM. OLETF rats are an animal model of type 2 DM with a metabolic syndrome and have pathological characteristics resembling those of human type 2 DM patients [8,9,10,11,12,35]. It was reported that 11-month-old OLETF rats may not reflect human DR, since there was no difference between the number of acellular capillaries in the retinas of normal rats and the number of acellular capillaries in the retinas of OLETF rats [36]. However, aging was found to change the retinas of OLETF rats, and Miyamura et al. [14] reported that ERGs in 14-month-old OLETF rats are similar to those seen in DM patients. In addition, it was reported that the vascular corrosion cast showed loop formations, tortuosity, narrowing, and caliber irregularity in the capillaries in the retina of 14-month-old OLETF rats. In our previous study using 15-month-old OLETF rats, we found that excess NO prevented CCO from having affinity for oxygen and caused a decrease in ATP levels due to CCO dysfunction in the retinas of OELTF rats [16]. Therefore, we selected 15-month-old OLETF rats to demonstrate the therapeutic effect of the Dis-NPs/ISG on DR. In this study, excessive food intake and water consumption were observed, and the blood test values for DM showed hyperglycemia and high triglyceride and cholesterol levels. The insulin levels were approximately 73 ng/dL and were lower than those in normal rats (103.5 ± 7.2 ng/dL, *n* = 8). These decreases in insulin levels reflect the progression of type 2 DM. Furthermore, the collapse of ATP production via NO was observed in the retinas of 15-month-old OELTF rats, and the a-wave amplitude, the b-wave amplitude, and the OP amplitude in the retinas of OLETF rats were lower in comparison with those in the retinas of normal rats. These results show that low ATP levels may induce the deterioration of ERGs.

In this study, the Dis-NPs/ISG was repeatedly instilled into the retinas of OLETF rats for 1 month (from 14 to 15 months of age), and the repetitive instillation of Dis-NPs/ISG was found to prevent NO production via iNOS mRNA and recover the decrease in ATP and the amplitude of ERGs (a-wave, b-wave, and OP amplitudes) in the 15-month-old OLETF rats. The therapeutic effect of retinal ATP dysfunction by instillation of Dis-NP/ISG was similar to that in oral administration of DSF (100 mg/kg body weight) [16]. Moreover, no difference in the iNOS mRNA, NO, and ATP levels were observed between of control LETO rats and OLETF rats instilled with Dis-NP/ISG (Figure 6). In addition, the instillation of Dis-NP/ISG can prevent the systemic side effects in comparison with oral administration [16], since the repetitive instillation of the Dis-NPs/ISG was found to not affect the food and water intake and some blood test values for DM in the OLETF rats (Figure 5). It was hypothesized that the collapse of ATP production due to CCO–NO binding caused the decrease in the a-wave, b-wave, and OP amplitudes in the retinas of 15-month-old OELTF rats. The repetitive instillation of the Dis-NPs/ISG recovered the deterioration of ERGs by inhibiting the iNOS expression in the retina.

## 5. Conclusions

We succeeded in preparing in situ gelling systems based on DIS nanoparticles and MC (Dis-NPs/ISG) and found that the Dis-NPs/ISG systems are effective ophthalmic DDSs for retinal drug delivery. In addition, we found that the collapse of ATP production due to excess NO caused a decrease in the amplitude of ERGs (a-wave, b-wave, and OP amplitudes) in the retinas of 15-month-old OELTF rats and that the repetitive instillation of the Dis-NPs/ISG recovered the deterioration of ERGs by inhibiting the iNOS expression in the retina. These findings provide important information that can be used to design further studies aimed at developing novel therapeutic approaches to DR.

## Figures and Tables

**Table 1:** Composition of ophthalmic disulfiram (DIS) formulations.

Formulation	DIS	BAC	Mannitol	HPCD	MC	Purified Water ad.	Treatment
Dis-sol	1 g	0.001 g	0.1 g	10 g	–	100 g	–
Dis-NPs	1 g	0.001 g	0.1 g	–	0.5 g	100 g	Bead mill
Dis-NPs/ISG	1 g	0.001 g	0.1 g	–	1.5 g	100 g	Bead mill
